# Editorial: Volume II: Structural and functional characterization of circular RNAs

**DOI:** 10.3389/fmolb.2022.1015990

**Published:** 2022-09-06

**Authors:** Tanvi Sinha, Kotb Abdelmohsen, Amaresh C. Panda

**Affiliations:** ^1^ Institute of Life Sciences, Bhubaneswar, India; ^2^ National Institute on Aging, National Institutes of Health (NIH), Baltimore, MD, United States

**Keywords:** circular RNA, RNA-sequencing, microRNA sponge, RNA-binding protein, gene regulation

Circular RNAs (circRNAs) are endogenously expressed covalently circularized RNA structures formed via non-canonical backsplicing of the primary transcript where the downstream donor site is ligated to the upstream acceptor site (Salzman et al., 2012; Jeck et al., 2013; Chen and Yang, 2015). Discovered more than 4 decades ago as “tiny circles” in plant viroids and later in eukaryotic cells, circRNAs were initially thought to be junk splicing byproducts (Sanger et al., 1976; Hsu and Coca-Prados, 1979; Nigro et al., 1991). However, later on, the identification and functional characterization of circRNAs gained momentum upon the advancements of transcriptome-wide RNA-sequencing technologies and novel circRNA annotation tools (Salzman et al., 2012; Memczak et al., 2013; Jakobi and Dieterich, 2019). Over the years, circRNAs have emerged as one of the most extensively studied RNA species across the eukaryotic tree of life. The unique chimeric closed loop form of circRNAs confers them with certain special properties such as enhanced tolerance to exonuclease attack rendering them stable compared to their linear isoforms (Salzman et al., 2012; Memczak et al., 2013). An ever-expanding body of research revealed that several circRNAs are conserved across species showing a nearly ubiquitous spatial- and temporal-expression pattern in myriads of mammalian cells and tissue types (Ji et al., 2019). Increasing evidence suggests that circRNAs can function as regulatory transcripts, which serve as microRNA and RNA-binding protein sponges, and directly act as translation templates (Panda, 2018; Das et al., 2021; Sinha et al., 2022).

Interestingly, the altered expression of circRNAs during development, disease progression, the presence in biofluids, and their extraordinary stability make them promising biomarkers for disease diagnosis and hence provide novel approaches for therapeutic intervention (Verduci et al., 2021; Chen et al., 2022). Understanding the unique structural and functional characteristics of circRNAs is essential to uncover gene regulatory systems in human development and disease progression (Chen et al., 2022). Although circRNAs have been established as important regulators of various pathophysiological processes, the molecular mechanisms of circRNAs in regulating gene expression remain to be explored. In addition, only a small fraction of circRNAs has been characterized for their biological functions from tens of thousands of circRNAs identified in humans and mice (Vromman et al., 2020). The goal of our Research Topic is to highlight the recent developments in circRNA research.

While it is crucial to elucidate circRNA function in normal cell physiology and biological processes, there is a rising interest in exploring the role of circRNA dysregulation in cancer, diabetes, neurological disorders, and other diseases. The review by Wang et al., provides a comprehensive summary of the biogenesis and functions of circRNAs, with a major emphasis on the potential molecular mechanisms and their therapeutic applications in urological cancers. This review article is instrumental in developing novel therapeutic strategies for urological cancers in the near future.

Recently thousands of circRNAs were identified in mouse and human skeletal muscle tissue. Moreover, a subset of highly abundant circRNAs was found in mouse skeletal muscle based on experimental validation by RNase R treatment, RT-qPCR analysis, and sequencing of the backsplice junctions. Examining plausible circRNA-miRNA-mRNA networks led to the discovery of conserved circular RNA *Nfix* (*circNfix*), which was found to interact with miR-204-5p miRNA, known to suppress myocyte enhancer factor 2c (MEF2C) expression, thereby promoting muscle cell differentiation, was reported by Das et al.

Major hurdles in circRNA research are their relatively low cellular abundance and the dependence of detection methods on input size and lacuna in downstream processing using conventional RNA-seq and computational methodologies. In this Research Topic, a targeted RNA sequencing approach known as Lexo-circSeq has been proposed by Naarmann-de Vries et al., which can detect nearly one hundred circRNAs and their linear counterparts in a single experiment as shown in total human heart RNA samples. Lexo-circSeq can be used to detect any specific circRNA in a large cohort of clinical samples, as demonstrated in hiPSC-derived cardiomyocytes, biopsies from patients diagnosed with hypertrophic cardiomyopathy and dilated cardiomyopathy. Thus, Lexo-circSeq is a cost-effective technique that can monitor circRNA expression patterns across large patient cohorts in terms of circular-to-linear ratios starting with a small input.

Another interesting article by Guria et al., demonstrated a novel template-dependent multiple displacement amplification (tdMDA)-NGS method. The improved-tdMDA-NGS method involves the typical rRNA depletion coupled with cDNA library preparation using H minus reverse transcriptase and RNase H treatment followed by tdMDA using Phi29 DNA polymerase. This cost-effective hybrid NGS approach improves the detection threshold to find even the marginally expressed circRNAs at lower NGS depths. Reportedly, there was a significant rise of nearly 11-fold in the total number of circular RNAs identified in rice plant and HeLa cell line using this improved-tdMDA-NGS method compared to the typical RNA-seq method of circRNA sequencing. Thus, the improved-tdMDA-NGS method can identify and characterize circRNAs in diverse biological samples.

This Research Topic covers the most recent findings concerning the roles of circRNA in health and diseases, as well as articles discussing improved state-of-the-art techniques to aid in accurately detecting circRNAs in clinically challenging scenarios. We believe that the articles highlighted in this Research Topic will be useful for carrying out a study to advance this emerging field of research.
